# Metabolomics profiling reveals different patterns in an animal model of asphyxial and dysrhythmic cardiac arrest

**DOI:** 10.1038/s41598-017-16857-6

**Published:** 2017-11-29

**Authors:** Dimitrios Varvarousis, Theodoros Xanthos, Giulio Ferino, Antonio Noto, Nicoletta Iacovidou, Massimo Mura, Paola Scano, Athanasios Chalkias, Apostolos Papalois, Fabio De-Giorgio, Alfonso Baldi, Paolo Mura, Chryssoula Staikou, Matteo Stocchero, Gabriele Finco, Ernesto d’Aloja, Emanuela Locci

**Affiliations:** 1 Medical School, National and Kapodistrian University of Athens, Athens, Greece; 2 European University Cyprus, Nicosia, Cyprus; 3 Department of Medical Sciences and Public Health, University of Cagliari, Cagliari, Italy; 4 Metabolic diseases Laboratory, Children Hospital “A. Cao”, Cagliari, Italy; 5 Department of Surgical Sciences, University of Cagliari, Cagliari, Italy; 6 Aretaieio Hospital, National and Kapodistrian University of Athens, Athens, Greece; 7 Department of Chemical and Geological Sciences, University of Cagliari, Cagliari, Italy; 8 Institute for the Study of Macromolecules, ISMAC, National Council of Research, Lab, NMR, Milan, Italy; 9 Hellenic Society of Cardiopulmonary Resuscitation, Athens, Greece; 10 Experimental-Research Center ELPEN Pharmaceutical, Athens, Greece; 11 Public Health Institute, Catholic University of Rome, Rome, Italy; 12 Department of Environmental, Biological and Pharmaceutical Sciences and Technologies, Second University of Naples, Caserta, Italy; 13 S-IN, Soluzioni Informatiche S.r.l., Vicenza, Italy

## Abstract

Cardiac arrest (CA) is not a uniform condition and its pathophysiology strongly depends on its cause. In this work we have used a metabolomics approach to study the dynamic metabolic changes occurring in the plasma samples of a swine model following two different causes of CA, namely asphyxia (ACA) and ventricular fibrillation (VFCA). Plasma samples were collected at baseline and every minute during the experimental phases. In order to identify the metabolomics profiles characterizing the two pathological entities, all samples were analysed by ^1^H NMR spectroscopy and LC-MS/MS spectrometry.The metabolomics fingerprints of ACA and VFCA significantly differed during the peri-arrest period and the resuscitation phase. Major alterations were observed in plasma concentrations of metabolites related to tricarboxylic acid (TCA) cycle, urea cycle, and anaplerotic replenishing of TCA. ACA animals showed significant metabolic disturbances during the asphyxial and CA phases, while for VFCA animals this phenomenon resulted shifted at the resuscitation phase. Interestingly, starting from the asphyxial phase, the ACA animals were stratified in two groups based on their metabolomics profiles that resulted to be correlated with the clinical outcome. Succinate overproduction was observed in the animals with the worse outcome, suggesting a potential prognostic role for this metabolite.

## Introduction

Cardiac arrest (CA) is a leading cause of death worldwide, affecting over 350,000 individuals every year^1,2^. However, CA is not a uniform condition and its pathophysiology strongly depends on the underlying cause. Asphyxial and cardiac causes count for the vast majority of CA. Evidence suggests that asphyxial CA (ACA) differs significantly from primary CA of cardiac origin (dysrhythmic) with regard to pathophysiological mechanisms, tissue damage, post-resuscitation organ dysfunction, and response to therapy^3^. ACA is characterized by a prolonged preceding period where hypoxia and acidosis progressively advance along with gradually deteriorating cardiopulmonary function until CA. On the contrary, dysrhythmic CA due to ventricular fibrillation (VF) or ventricular tachycardia (VT) leads to sudden and complete cessation of blood flow. Despite the important advances in our understanding of CA and cardiopulmonary resuscitation (CPR) over the last decades, the pathophysiological mechanisms underlying CA and the post-resuscitation period are only partially understood and their complete elucidation remains a challenge.

Metabolomics refers to the identification and the quantification of the low molecular weight metabolites (the metabolome) present in biological fluids or tissues, allowing the study of dynamic metabolic alterations of a living system in response to genetic modifications or physiopathological stimuli^4^. Metabolomics employs mainly two analytical techniques based on proton Nuclear Magnetic Resonance (^1^H NMR) spectroscopy, and Mass Spectrometry (MS) coupled to either Gas Chromatography (GC) or Liquid Chromatography (LC). Univariate and multivariate statistical analysis are performed to unravel the hidden information contained in the collected data^5^.

Several recent studies proved metabolomics to be a powerful tool for monitoring hypoxic/ischemic events, and several metabolites have been suggested as potential biomarkers^6–9^. Metabolomics could be of great advantage in establishing metabolic signatures in experimental models of CA, where metabolic changes occur and advance rapidly^10^. Plasma metabolite concentration changes provide useful information, as they are the closest link to cellular metabolism in the whole body and to its disturbances following CA and/or resuscitation. A better understanding of the alterations in metabolites over time may be extremely helpful in the recognition of the underlying pathophysiological mechanisms. Moreover, identifying specific plasma metabolite changes, associated with responses either to asphyxial or dysrhythmic CA, may represent a feasible tool to achieve an earlier diagnosis and a more accurate prognosis.

The aim of this study was to investigate in an animal model the differences that exist between asphyxial and dysrhythmic CA, concerning hemodynamic parameters and metabolic status, in the arrest phase, during cardiopulmonary resuscitation and in the post-resuscitation period. Plasma metabolic modifications were investigated by means of high resolution ^1^H NMR and LC-MS/MS coupled with univariate and multivariate statistical analysis. In particular, NMR was used to obtain a global metabolomics profile of the plasma samples and LC-MS/MS was used to exactly quantify a selected set of key metabolites, such as amino acids, acylcarnitines, Krebs cycle intermediates, involved in cellular energy metabolism and in amino acid metabolism/catabolism. Thus, the identification of potential biomarkers, useful to distinguish between the two etiologies, was addressed.

## Results and Discussion

### Clinical and hemodynamic parameters

**Figure 1:**
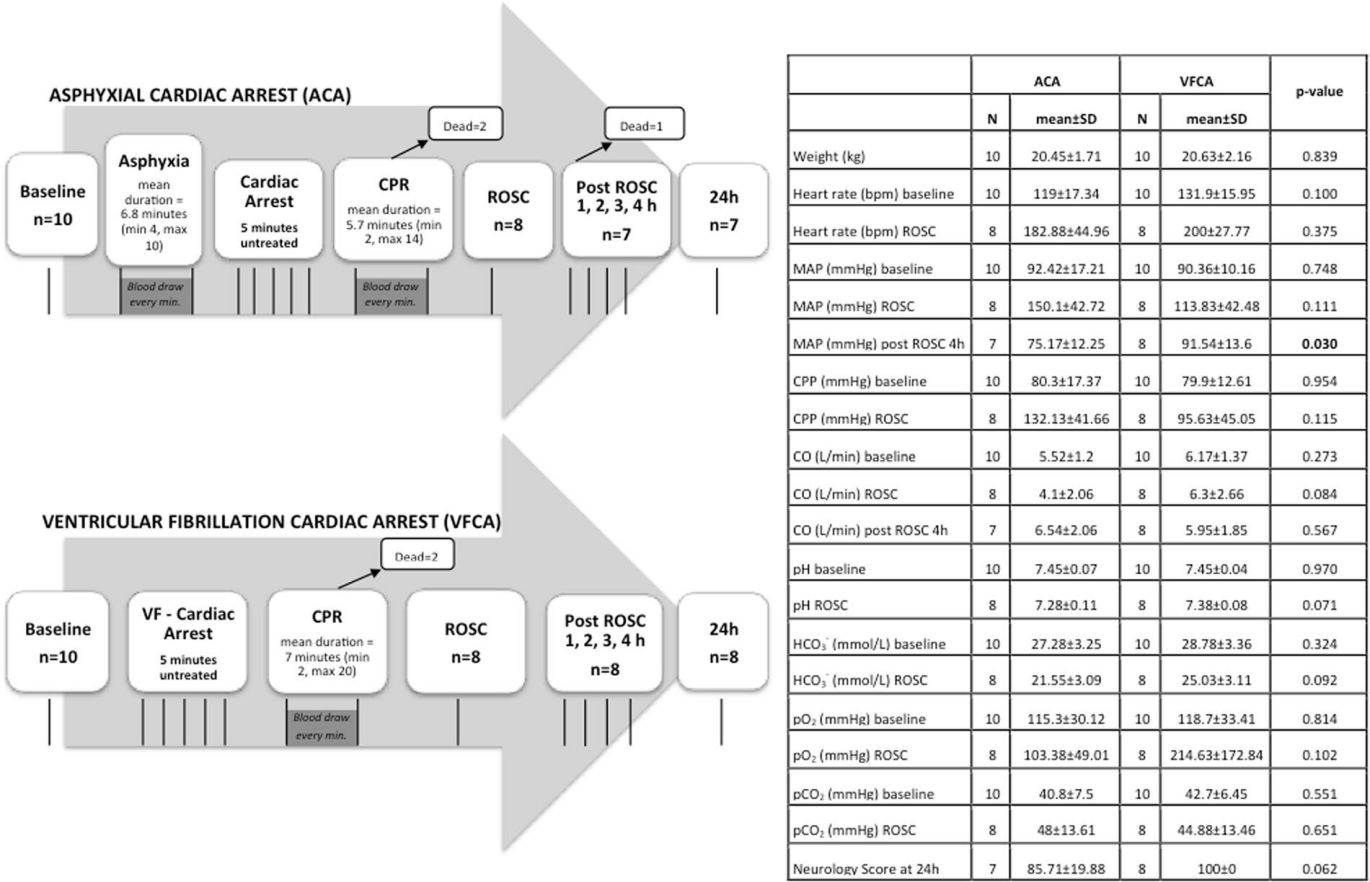
Overview of the experimental study design and summary of the clinical/hemodynamic parameters. Clinical/hemodynamic parameters (weight, heart rate, MAP, CPP, CO) and arterial blood gas analyses of animals of the ACA and VFCA group at baseline, ROSC and 4 h post-ROSC, along with the p-values for the intergroup comparison calculated using the Student’s t-test. Data are presented as mean ± SD. Vertical lines in the scheme indicate blood draws. ACA = asphyxial cardiac arrest group; VFCA = ventricular fibrillation cardiac arrest group; ROSC = return of spontaneous circulation; MAP = mean arterial pressure; CPP = coronary perfusion pressure; CO = cardiac output; SD = standard deviation; bpm = beats per minute; pO_2_ = arterial oxygen tension; pCO_2_ = arterial carbon dioxide tension.

The overview of the experimental study and the summary of the clinical and hemodynamic parameters are reported in Fig. 1. The animals were randomized into two groups and asphyxial or dysrhythmic CA was induced. For the ACA group, the duration of the pre-arrest period (asphyxia) differed between 4 and 10 minutes (mean value 6.8 minutes). 4 animals had an asphyxial time ≤ 5 minutes, while for the rest of the ACA group (n = 6) asphyxial time was relatively longer (≥7 minutes). For animals that achieved Return of Spontaneous Circulation (ROSC), mean CPR-to-ROSC duration was 3.4 minutes for the ACA group versus 4.25 minutes for the VFCA group.

Overall survival rate after 24 h was 70% in the ACA and 80% in the VFCA group. In the ACA group, during asphyxia, mean arterial pressure (MAP) of animals showed a significant drop after the 4th minute (p-value < 0.005).

There were no statistically significant differences in the hemodynamic parameters [heart rate, mean arterial pressure (MAP), coronary perfusion pressure (CPP) and cardiac output (CO)] between the ACA and VFCA groups during the experiment, but lower values of CO and MAP were observed at ROSC and during the post-resuscitation period for the ACA group.

### Metabolomics analyses

**Figure 2:**
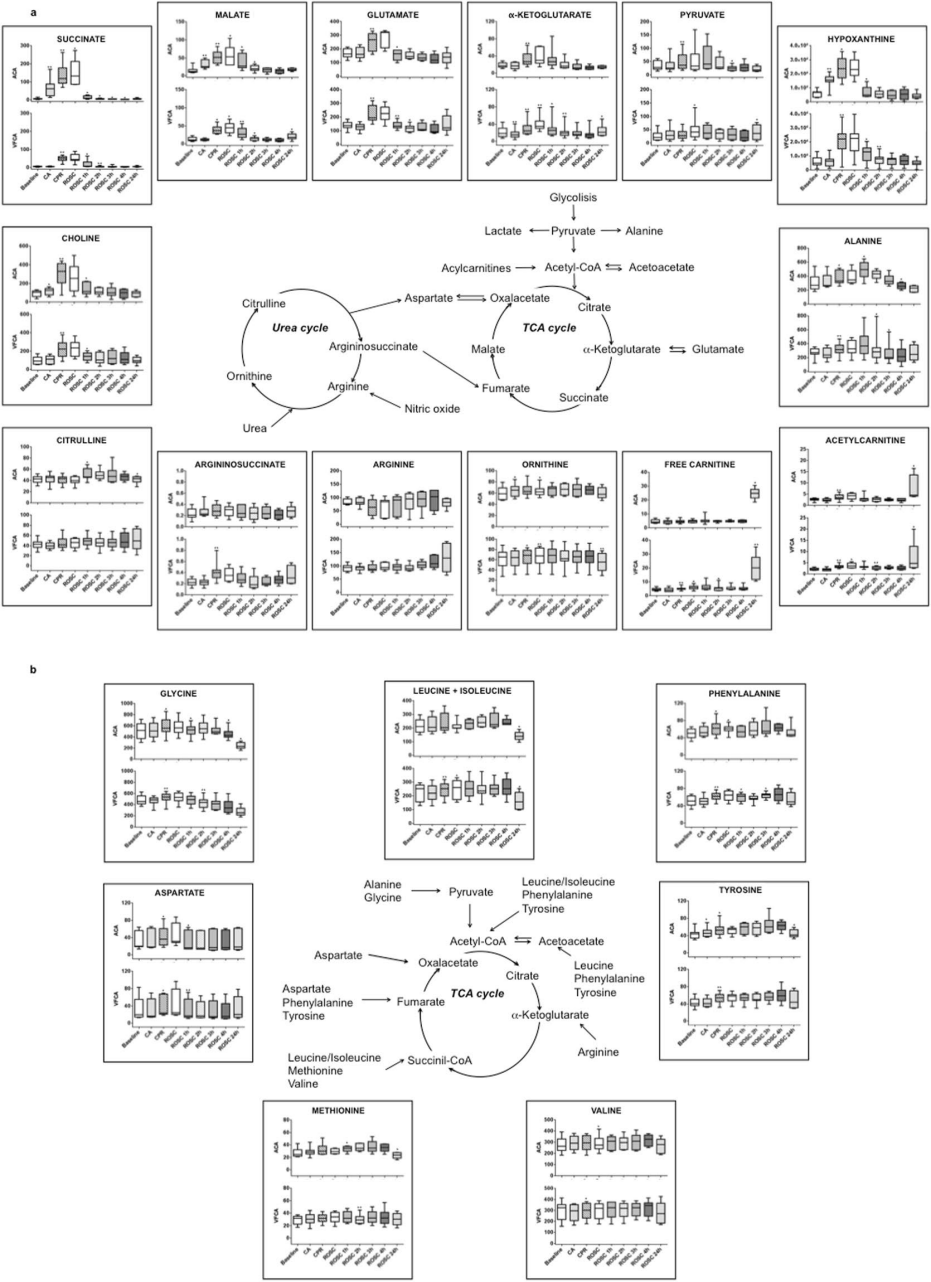
Overview of LC-MS/MS quantification of key metabolite changes in plasma samples of animals undergoing asphyxial and dysrhythmic CA. Results, expressed in µmol/L, are graphed as box plots, indicating median values and upper and lower quartile distributions, at baseline, CA (last minute), CPR (last minute), ROSC and post-ROSC (1, 2, 3, 4, 24 h). (**a**) TCA cycle, urea cycle, lipid metabolism intermediates. (**b**) Anaplerotic replenishing of TCA cycle. Asterisks indicate statistical significance regarding the within group comparison of each phase with the previous one calculated by paired Wilcoxon t-test (*p-value ≤ 0.05, **p-value ≤ 0.01, ***p-value ≤ 0.001).

Plasma metabolomics analyses were performed on 20 landrace/large-white female pigs exposed either to ACA (n = 10) and VFCA (n = 10). A total of 380 plasma samples, withdrawn during the different phases of the experiment (Fig. 1), were analysed. Global untargeted plasma profiles were obtained by ^1^H NMR spectroscopy and spectral data were submitted to multivariate statistical analysis in order to identify changes in circulating metabolites during the different phases of the experiment and to compare the profiles associated with the two CA models. LC/MS-MS was used for the exact quantification of a chosen set of key metabolites, which are involved in energy production and in amino acid metabolism/catabolism. Figure 2 reports an overview of the LC-MS/MS data; in particular it depicts the tricarboxylic acid (TCA) cycle, urea cycle, lipid metabolism and anaplerotic replenishing of the TCA cycle intermediates modifications during the experiment. Due to presence of an exogenous contaminant, the spectral data of one ACA animal were excluded from statistical analysis.

All VFCA animals homogeneously responded after the onset of CA. In the ACA group we were able to identify two subgroups of animals regarding their metabolomics response in association with poor neurological outcome and death. Specifically, ACA animals that did not achieve ROSC (n = 2), or died early after ROSC (n = 1) or had a final neurology score (NS) < 70 at 24 h (n = 1), were classified as ‘damaged’ animals. The remaining ACA animals (n = 6) were classified as ‘no-damaged’ animals.

No significant differences were observed between the two groups at baseline, after stabilization of the animals. This indicates that the basal metabolomics profile is very similar for all the animals, a mandatory condition to compare the metabolic perturbations following the two different CA mechanisms.

### The effect of cardiac arrest on the metabolome

**Figure 3:**
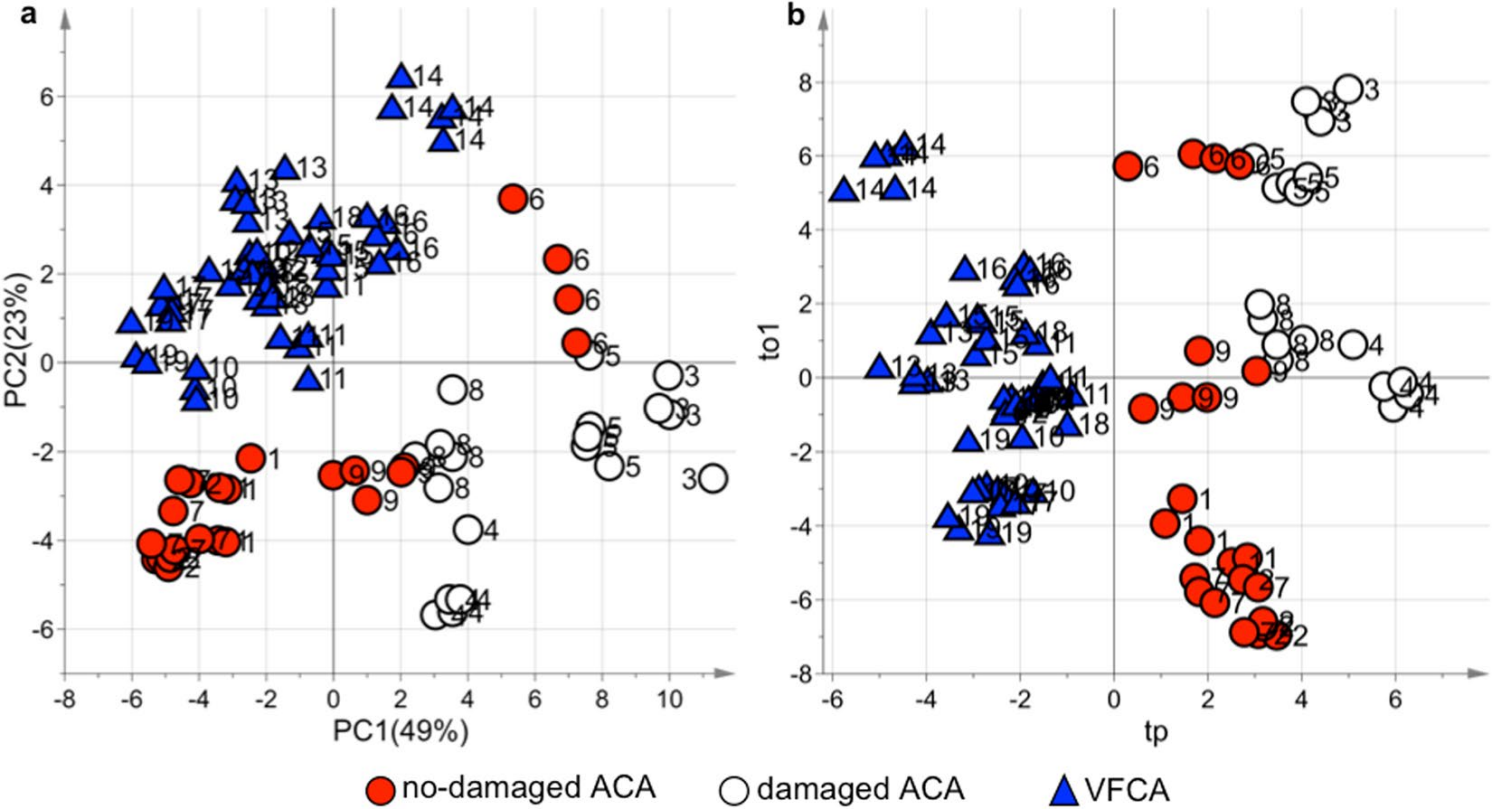
Comparative analysis of plasma samples during CA. Score scatter plots of the (**a**) PCA and (**b**) ptPLS2-DA models of ACA and VFCA plasma samples during the five minutes of CA (one samples per minute). No-damaged ACA samples are represented by red circles, damaged ACA samples by empty circles, and VFCA samples by blue triangles. Labels indicate the animal number.

ACA and VFCA animals were compared by analysing the samples collected during the five minutes of untreated CA (one sample per minute). The score scatter plots of PCA and ptPLS2-DA models obtained for the ^1^H NMR data set are shown in Fig. 3. ACA and VFCA samples are well separated in the PCA (Fig. 3a, explained variance by the first two components 72%), indicating that they are characterized by different metabolic profiles. The repeated measurements of samples collected from the same animal are close to each other proving that the intra-animal time variance is less than the variance between the two CA models. Concerning the ptPLS2-DA (Fig. 3b), the design matrix, including time and CA cause, was used as response to drive PLS regression. The model showed A = 1 + 2 components, R^2^ = 0.84 (p-value < 0.001) and Q^2^ = 0.82 (p-value < 0.001) being the time effect not significant (p-value = 0.22). The predictive component (tp), explaining the differences between ACA and VFCA, is reported as x-axis and the first orthogonal component as y-axis (to1). The ACA samples have positive tp values whereas the VFCA samples have negative ones, further underlying that they have different metabolic profiles. In both models, the damaged ACA animals, represented with a different symbol only for descriptive purposes, lie in the outer part of the plot, suggesting an intragroup stratification of these samples.

The discriminant metabolites, responsible for the separation between ACA and VFCA samples, arising from the analysis of the ^1^H NMR and LC-MS/MS data by multivariate data analysis and mixed-effect modelling, are reported in Table 1. Significantly higher levels of lactate, succinate, malate, fumarate, glutamate, hypoxantine, uridine, and cytidine were detected in ACA samples. Conversely, VFCA did not result in any significant alteration of the metabolomics signature. This constitutes an intriguing finding, which is related to the higher degree of tissue hypoxia and acidosis characterizing the ACA group. The fact that the ptPLS2-DA model indicated no time-related differences within the five minutes of CA is consistent with a no-flow condition. Only hypoxanthine and lactate showed a time-related behaviour^6^.

**Table 1:** Metabolites significantly accumulated in the ACA compared to VFCA samples, with corresponding p-values from mixed-effects models, considering both group effect (ACA and VFCA) and time effect. Data analysis was applied to ^1^H NMR and LC-MS/MS data.

Cardiac arrest				
Metabolite	Intercept (p-value)	Time (p-value)	Group (p-value)	R^2^
Lactate	23 (<0.001)	0.33 (0.02)	−12 (<0.001)	0.98
Succinate	67 (<0.001)	3 (0.09)	−72 (<0.001)	0.99
Malate	32 (<0.001)	0.45 (0.46)	−22 (<0.001)	0.96
Fumarate	0.002 (0.04)	0.00007 (0.70)	−0.002 (0.03)	0.40
Glutamate	173 (<0.001)	−0.79 (0.79)	−47 (0.01)	0.97
Hypoxanthine	15000 (<0.001)	790 (0.004)	−9700 (<0.001)	0.94
Uridine	−0.02 (<0.001)	0.00001 (0.97)	−0.0008 (0.004)	0.75
Cytidine	0.003 (0.30)	−0.001 (0.10)	−0.008 (0.008)	0.23

### The effect of asphyxia on the metabolome

**Figure 4:**
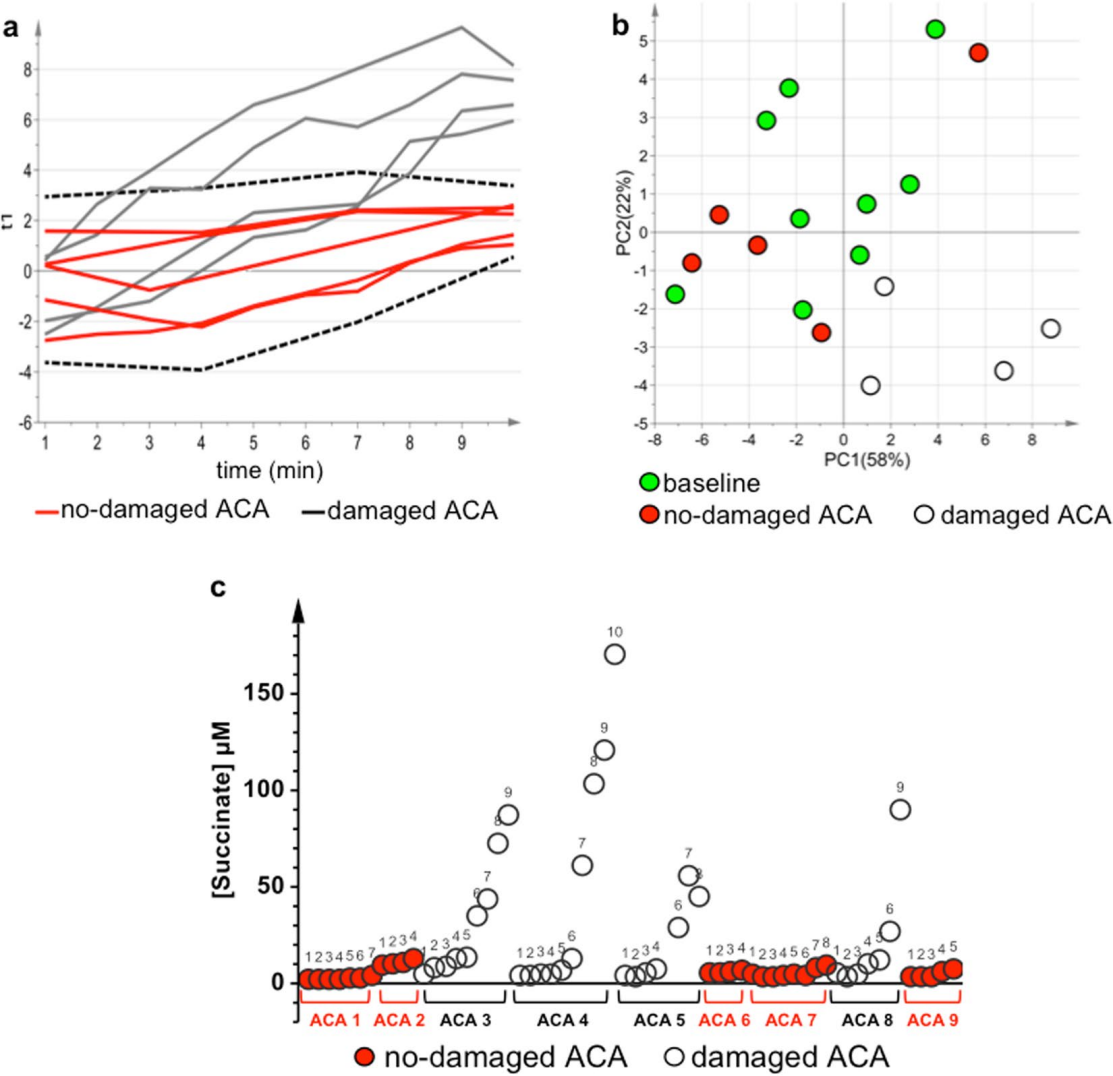
The effect of asphyxia on the plasma metabolome of ACA animals. (**a**) Chart for the BSPC model obtained for no-damaged ACA animals during the asphyxial period. The dashed lines correspond to the limit of 2 standard deviations. The trajectories of the no-damaged animals are represented with red lines, while the trajectories of the damaged animals with grey lines. (**b**) PCA score scatter plot of plasma samples belonging to ACA animals at baseline and at the last minute of the asphyxial period. The explained variance of the first two components is 80%. Baseline samples are represented by green circles, no-damaged ACA samples by red circles, and damaged ACA samples by empty circles. (**c**) Modifications of succinate concentration over the asphyxial period for each ACA animal reported in the x-axis. Numerical labels correspond to the asphyxial minute. No-damaged ACA samples are represented by red circles, and damaged ACA samples by empty circles.

The asphyxial period duration was highly variable from one animal to the other, ranging from 4 to 10 minutes. In order to analyse the asphyxia-induced metabolic perturbations over time a multivariate Batch Statistical Process Control (BSPC)^11^ approach was applied. A univocal timescale was obtained by linear expansion of the asphyxial period to 10 minutes for all the animals. The PLS model showed A = 3 components, R^2^ = 0.71 (p-value < 0.001) and Q^2^ = 0.48 (p-value < 0.001). Figure 4a shows the chart representing the individual metabolomics trajectory plotted versus time of each ACA animal. The dashed lines correspond to the limit of 2 standard deviations. The trajectories of the no-damaged animals (red lines) lie within this region, while those belonging to the damaged animals (grey lines) exceed these limits, moving constantly away from those of the no-damaged animals during this period. Major metabolite modifications occurring during this phase are reported in Table 2. As can be seen, almost all metabolites increased with time in both ACA groups, but more significantly in the damaged one. This is also evident in the unsupervised PCA model of samples at baseline and at the last minute of the asphyxial period (Fig. 4b). As shown, the samples belonging to no damaged animals are randomly distributed with baseline ones, while the samples of damaged animals form a separate cluster located in the lower right hand side region.

**Table 2:** Metabolites significantly modified during the asphyxial period identified by mixed-effects models (linear model with interaction), considering both group effect (ACA damaged and no- damaged) and time effect, with corresponding p-values. Data analysis was applied to ^1^H NMR and LC-MS/MS data.

Asphyxial period							
Metabolite	Significant variation in time		Intercept (p-value)	Time (p-value)	Group (p-value)	Time*group (p-value)	R^2^
	damaged ACA	no-damaged ACA					
Argininosuccinate	↑	—	0.22 (0.003)	−0.0002 (0.9)	−0.03 (0.7)	0.013 (0.003)	0.82
Succinate	↑	—	4.1 (0.5)	0.3 (0.8)	−16 (0.04)	8 (<0.001)	0.77
Ornithine	↑	—	61 (<0.001)	0.2 (0.3)	−0.7 (0.9)	0.5 (0.046)	0.98
Glycine	↑	—	522 (<0.001)	1 (0.3)	−26 (0.8)	4 (0.03)	0.97
Glutamate	↑	↓	170 (<0.001)	−2 (0.06)	−38 (0.1)	10 (<0.001)	0.79
Aspartate	↑	—	36 (0.01)	−0.3 (0.3)	−10 (0.5)	0.9 (0.04)	0.95
Tyrosine	↑	—	38 (<0.001)	0.1 (0.4)	8 (0.2)	0.7 (0.03)	0.87
Phenylalanine	↑	—	44 (<0.001)	0.4 (0.07)	9 (0.1)	0.8 (0.009)	0.91
Leucine + Isoleucine	↑	—	204 (<0.001)	0.6 (0.3)	24 (0.5)	1.8 (0.04)	0.95
Acetylcarnitine	↑	—	2.4 (<0.001)	−0.03 (0.2)	−0.3 (0.5)	0.14 (0.006)	0.84
Free carnitine	↑	—	5 (<0.001)	0.005 (0.8)	−0.62 (0.4)	0.08 (0.01)	0.90
Hypoxanthine	↑	—	4612 (0.003)	279 (0.2)	−1839 (0.3)	1211 (0.004)	0.82
α-Ketoglutarate	↑	↓	19 (<0.001)	−0.5 (0.05)	−6 (0.3)	1.1 (0.009)	0.87
Malate	↑	—	13 (0.001)	0.4 (0.2)	−4.4 (0.2)	2.7 (<0.001)	0.90
Lactate	↑	—	19 (0.002)	0.79 (0.008)	−9 (0.02)	—	0.95

↑↓ indicate if the metabolite increased or decreased in time, respectively.

Asphyxia induces global, progressive hypoxia and gradual ATP depletion^3,12^. A gradual accumulation of TCA cycle intermediates, being TCA cycle and electron transport chain^13^ impaired by shortage of oxygen, and of lactate, indicating activation of anaerobic glycolysis, was detected in the plasma of asphyxiated animals. Moreover, in the damaged animals, a peculiar increase of TCA cycle intermediates and by-products of all TCA anaplerotic cycles was observed along with modifications in the urea cycle, purine and protein catabolism (Table 2). Interestingly, glutamate and α-ketoglutarate decreased in time in the no-damaged animals. At the end of the asphyxial period, regardless of its duration, ACA damaged and no-damaged plasma samples were characterized by a different metabolic profile (Supplementary Fig. S1 and Supplementary Table S1).

During progressive hypoxia, the respiratory chain function is switched from oxidation of NAD-related substrates to succinate oxidation^13^. Interestingly, we observed during the pre-arrest period different trends of succinate concentration in animals undergoing asphyxia. More specifically, during asphyxia progression, overproduction of succinate, up to 40-folds with respect to the baseline, was only observed in damaged animals, while a slight increase (up to 3-folds) was observed in no damaged ones (Fig. 4c). During the pre-arrest period there is a unique situation, in which there is still blood flow but with progressive shortage of oxygen. The different concentrations of succinate observed in blood may be related to the ‘reverse’ cellular activity to reduce fumarate to succinate and, finally, to produce reactive oxygen species (ROS)^14^. The higher the plasma concentration of succinate, even in the pre-arrest period, the worst the outcome. Succinate links the TCA cycle to the respiratory chain because succinate-coenzyme Q reductase is an enzyme-complex directly linked to the electron transport chain. General de-energization and mitochondrial complexes inactivation take place in parallel with oxygen shortage until complete decompensation^13^. Succinate, which reflects the ability of the cell to cope with the excess of reducing equivalents, accumulates during hypoxia in cells, in tissues, and eventually in blood^6–9^. Formation of endogenous succinate in hypoxia is tissue-specific and, in our model, seems to be based on aspartate and glutamate aminotransferase reactions, α-ketoglutarate phosphorylation, and ‘reverse’ TCA fluxing of carbon skeletons from aspartate (TCA cycle reversal)^14^. Recently, an unifying mechanism for mitochondrial superoxide production during ischemia and reperfusion injury was proposed, suggesting the pivotal role of fumarate/succinate in the hypoxic/ischemic cellular response^14^. Accordingly, hypoxia/ischemia induces the reverse activity of complex II (succinate-coenzyme Q reductase or succinate dehydrogenase) leading to a progressive reduction of fumarate to succinate. In a progressive oxygen shortage condition, fumarate acts as the final acceptor of electrons yielding to the accumulation of succinate, which may act, mainly depending on its concentration, as a pro-survival or pro-death signalling system. Our experiment documented a pathological increase of succinate in the very early stage of asphyxia, prior to arrest, which in the damaged animals may be responsible, due to the residual presence of oxygen and flow, of the cell damage driven by RET-mediated ROS production trough complex I^15^. This finding suggests a putative role of succinate as potential prognostic marker of the hypoxic insult severity and of the clinical outcome.

Interestingly, in our experimental group – even if limited in numbers – two no-damaged animals (20%) showed a 7–8 minutes asphyxial period, challenging the hypothesis of an inverse correlation between the duration of asphyxia and the survival rate. On the other hand, all the damaged animals showed an asphyxial time-to-arrest longer than 7 minutes.

Hypoxanthine, a metabolite of purines, is also accumulated during hypoxia, due to gradual ATP reduction^16^. Accordingly, in our experiment, a significant increase in hypoxanthine concentration was observed with progression of asphyxia, and this trend kept going on even during the five minutes of CA. Hypoxanthine may act as substrate for the formation of ROS during early asphyxial phase and re-oxygenation, resulting in local and systemic oxidative stress through widespread lipid perioxidation^17^.

The increase in the urea cycle intermediates, namely argininosuccinate and ornithine, observed in our study, reflects either an up-regulation of the urea cycle activity or an impaired cycle function^18,19^. In particular, argininosuccinate may be involved in the TCA cycle via fumarate. Glutamate originates from glutaminolysis, and may enter the TCA cycle via α-ketoglutarate, although extracellular glutamate can also be related to the disturbance of the ionic homeostasis of the cell membranes and to excitotoxicity since in normal conditions glutamate is converted into glutamine by astrocytes^20^. Tyrosine and phenylalanine, which are by-products of the proteolysis, may act as TCA or acetyl-CoA precursors^21^. Additionally, acetone and 3-hydroxybutirate are alternative sources for energy production^8,19,22^. Uridine is a precursor of brain membrane phospholipids in the form of UTP^23^. Its increase may be related to the damage of membrane lipid components or to an attempt to maintain brain metabolism during asphyxia, as the catabolism of pyrimidines produces TCA cycle intermediates. Free carnitine and acetylcarnitine increased during progressive hypoxia, acting as a protective mechanism on the energy process^6,22,24^.

### The effect of cardiopulmonary resuscitation on the metabolome

**Figure 5:**
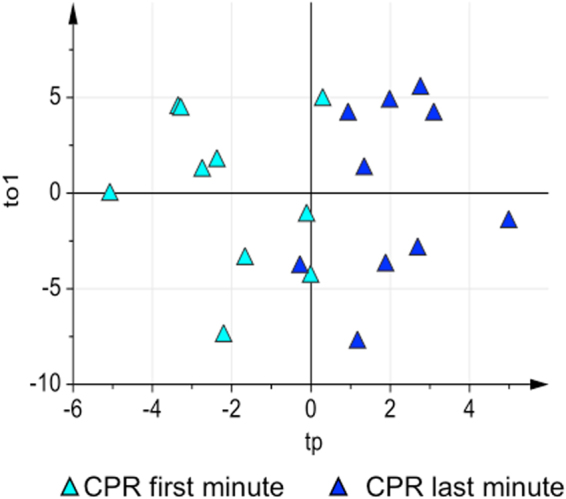
The effect of CPR on the plasma metabolome of VFCA animals. Score scatter plot of the ptPLS2-DA model of VFCA plasma samples at the beginning (turquoise triangles) and at the end (blue triangles) of CPR.

Effective CPR is based on continuous high quality chest compressions along with 100% oxygen ventilation. The goal of this ‘low-flow’ phase is an early ROSC. In our study, CPR duration was highly variable, from 2 to 20 minutes. In surviving animals CPR varied from 2 to 8 minutes, while in no-surviving animals a 10 minutes threshold was adopted for the data analysis, since after this time point no major metabolic changes were observed. ACA and VFCA metabolomics profiles were different at the beginning (first minute) and at the end (last minute) of CPR (Supplementary Fig. S2 and Supplementary Table S2). Concerning the ACA group, the profiles of damaged and no-damaged animals remained well separated both at the beginning and at the end of at CPR (Supplementary Fig. S3 and Supplementary Table S3). Finally, in the VFCA group, where up to now no significant variations were observed, major metabolic pathways derangement occurred. Figure 5 shows the ptPLS2-DA model of VFCA samples at the beginning and at the end of CPR [A = 1 + 1 components, R^2^ = 0.67 (p-value = 0.004), Q^2^ = 0.45 (p-value = 0.002)]. The corresponding metabolic modifications are reported in Table 3.

**Table 3:** Metabolites significantly accumulated during CPR in VFCA samples, with corresponding p-values and q-values for the Wilcoxon test applied to ^1^H NMR and LC-MS/MS data, and with mean, minimum and maximum fold change increase.

CPR in VFCA				
Metabolite	p-value	q-value	Mean Fold Change	Min-Max Fold Change
Lactate	0.04	0.12	0.53	0.01–1.52
Succinate	0.002	0.01	5.39	0.25–10.35
Malate	0.006	0.03	2.11	0.01–3.40
α-ketoglutarate	0.006	0.03	0.86	0.21–2.53
Hypoxanthine	0.002	0.01	1.92	0.09–6.02
Glutamate	0.004	0.02	0.55	0.09–1.15
Argininosuccinate	0.002	0.01	1.51	0.31–7.97
Ornithine	0.06	0.15	0.08	0.01–0.20
Aspartate	0.03	0.08	0.57	0.01–2.71
Choline	0.002	0.01	1.14	0.19–2.83
Alanine	0.01	0.05	0.32	0.15–0.71
Free carnitine	0.002	0.01	0.32	0.07–1.01
Acetylcarnitine	0.01	0.05	0.61	0.01–1.28
Methionine	0.01	0.05	0.15	0.01–0.31
Phenylalanine	0.01	0.05	0.27	0.05–0.50
Tyrosine	0.004	0.02	0.31	0.07–0.69
Glycine	0.01	0.05	0.18	0.01–0.40
Inosine	0.03	0.08	1.33	0.01–3.71
Uridine	0.02	0.08	2.22	0.01–5.07
Cytidine	0.03	0.08	0.52	0.01–2.72

At the beginning of CPR our study identified a further increase of lactate, succinate, malate, glutamate, and in a less evident way of alanine (p-value = 0.08) in the plasma of ACA animals when compared to VFCA (Supplementary Table S2). At the end of CPR, lactate and succinate remained more expressed in ACA together with inosine, while arginine (p-value = 0.08) resulted higher in VFCA. Taking into consideration the metabolic modifications occurring within each group separately, it is possible to observe that, within the ACA group (Supplementary Table S3), the comparison between damaged and no-damaged animals, both at the beginning and at the end of CPR, indicated that TCA cycle intermediates and hypoxanthine kept increasing together with glutamate, 3-hydroxybutyrate, acetylcarnitine, tyrosine, and phenylalanine (Supplementary Table S3). Interestingly, the presence of fumarate highly suggests the return of complex II to the forward activity in the mitochondrial inner membrane, due to the presence of oxygen that induces the oxidation of succinate accumulated during the hypoxic/ischemic event^15^. In VFCA, the comparison between the beginning and the end of CPR indicated the significant accumulation of lactate, TCA cycle intermediates, urea cycle intermediates, anaplerotic replenishing TCA cycle intermediates and some amino acids. In contrast with ACA, after VFCA, initiation of CPR is required for the metabolic modifications to be detected in plasma, and this shifted phenomenon, although expected, is a considerable feature to distinguish the two CA insults. The fact that, at the end of CPR, succinate and lactate continued to be elevated in a higher degree in ACA compared to VFCA, and that fumarate is only represented in the ACA group, highlights the important role of the asphyxial pre-arrest period and the impact of hypoxia on mitochondria.

Our results are in line with those reported in previous similar studies investigating resuscitation after asphyxia^7–9^. In contrast, another experimental study focused on resuscitation of asphyxia using different re-oxygenation protocols reported decline of the particular metabolites during the resuscitation phase^6^. Nevertheless, none of these mentioned experimental studies investigated complete derangement to CA.

A significant rise in alanine and aspartate plasma concentration was observed during on-going CPR only in the VFCA group. Their progressive increase during tissue re-oxygenation may be possibly explained by a univocal flow from pyruvate to alanine, which is supposed to serve for aspartate and glutamate production *via* transamination^25^. The increase of free plasmatic alanine could have a role, as for all other free amino acids, in fuelling the TCA cycle *via* pyruvate. Glutamate and aspartate are excitatory amino acids involved in many important transamination reactions, and they can be used as ‘fuel’ for the TCA and the urea cycle. Alternatively, the important elevation in plasma concentration of glutamate, during on-going re-oxygenation efforts, may be related to the brain damage as previously discussed.

The urea cycle intermediates modifications during resuscitation deserve special mention. Argininosuccinate and, in a less evident way, ornithine increased during CPR in VFCA group. At the same time, arginine decreased during on-going resuscitation of (no-damage) ACA animals, contrary to VFCA where there was no significant dynamic change. This finding indicates differences in activation of the urea cycle during CPR of ACA and VFCA, taking also into consideration other important effects of these metabolites. For example, arginine participates in NO production by endothelial NO synthase and this might occur in a higher degree during on-going resuscitated ACA^18,19,26,27^. In turn, NO generation promotes vasodilation, in order to compensate the existing pronounced tissue hypoxia after ACA. Partial deactivation and reactivation of the urea cycle enzymes during resuscitated ACA and VFCA animals might explain the corresponding differences in argininosuccinate changes, which, of note, was found significantly increased only in resuscitated VFCA animals. This could also be attributed to the significant increase in succinate, which at re-oxygenation returns to be oxidized to fumarate, inhibiting argininosuccinate further metabolism to fumarate.

### The post-resuscitation period

Our experimental protocol involved a 4h-monitoring period after ROSC, reflecting the early post-resuscitation phase. During this period, some differences in hemodynamics were observed, such as lower values of MAP and CO for the ACA group. In addition, a worse degree of metabolic acidosis after ACA was indicated by the arterial blood gas analyses (Fig. 1). Another very interesting observation was that all ACA animals, in which duration of CPR was more than 2 minutes, either did not survive or had a functional neurologic deficit at 24 h post-ROSC.

After ROSC, the reversal of all metabolic alterations is expected because of mitochondrial recovery. In this study, irrespectively of the CA cause, most metabolic disturbances showed a trend towards normalization within the first hour after ROSC (Supplementary Fig. S4 and Supplementary Table S4). A progressive decrease in the levels of succinate, malate, glutamate, and hypoxanthine was observed in both groups. During the first 4 hours after ROSC, the metabolomics profiles of ACA and VFCA samples progressively changed still maintaining a clear difference in their trajectories (Supplementary Fig. S5). After 24 hours, the metabolomics profiles of ACA and VFCA were not distinguishable anymore, although there were still differences with respect to baseline (Supplementary Fig. S5).

The significant elevation in plasma fatty acid mobilizing carnitines at 24 hours after ROSC (Fig. 2), irrespectively of ACA or VFCA, is consistent with findings of other metabolomics studies investigating mainly resuscitation of asphyxial insults^6,22,24^.

### Potential biomarkers identification

Recognition of several metabolic profiles and assessment of their dynamic changes over time during ACA and VFCA was one of the objectives in our study. We further sought to investigate whether specific key metabolites can possibly be correlated with the cause or with the prognosis of CA. Significant differences were observed in succinate and hypoxanthine levels between ACA and VFCA animals during CA and CPR, with markedly elevated concentrations after ACA. These metabolites increased during asphyxia and they have been identified in several recent studies as possible markers of hypoxia^16^. During untreated VFCA (no-flow phase) there were even no changes in plasma concentration of these particular metabolites, until initiation of CPR. Thus, we can assume that succinate and hypoxanthine could provide important clinical information in cases of out-of-hospital CA victims or in forensic medicine and be useful in identifying the cause of CA, although further extensive assessment and validation is needed. Moreover, relatively recent evidence suggests additional roles for some metabolites such as succinate in a hormone-like manner, once released into the circulation. A protective effect has been related to the regulation of NO synthesis and metabolism^28^. Succinate may have further actions, in the context of adaptive mechanisms to meet metabolic demands, even though it remains unclear whether they are protective or detrimental^10^. In ACA animals, the marked succinate overproduction during the pre-arrest phase seemed to be responsible for the worse outcome, regardless of the duration of asphyxia. In fact, succinate accumulation seems to be linked to the production of mitochondrial ROS following ischemia^14^. In particular at reperfusion, the oxidation of the accumulated succinate induces the reverse electron transport at complex I that drives ROS production and mitochondrial disruption that causes apoptotic and necrotic cell death. The animals that showed a good outcome, despite the relatively long asphyxial period, were characterized by succinate concentrations close to the baseline values; this further suggests a prognostic role of succinate, especially during asphyxial insults.

### Limitations of the study

This was an experimental study comparing directly ACA and VFCA, while no sham control group was used in order to exclude possible effects of anesthesia on the plasma metabolome. Moreover, the choice of the 5 minutes duration of the untreated CA phase, which could be considered as relatively short, was based upon the expectation of a higher ROSC and survival rate at 24 h, in order to better investigate the post-resuscitation period. From this point of view, we cannot exclude possible more intense metabolic changes during the arrest phase, but to this aim a longer no-flow state should be induced. Finally, this is a descriptive study, focusing on the hemodynamic and metabolomics modifications during all the experimental phases, going from baseline to 24 hours post-ROSC. For these reasons, no univocal mechanism can be drawn by our laboratory data and complementary studies (e.g. gene expression, functional analyses, etc.) are needed to unravel the complex pathways activated by CA. The lack of functional data clearly limits the clinical utility of these findings.

## Conclusion

To the best of our knowledge, this is the first experimental study allowing a close view of the metabolic pathways that are activated in response to CA and CPR. We reported a comprehensive characterization of the metabolomics profiles occurring during the multifactorial nature of these circumstances and that have remained largely unknown until now. We presented evidence showing that the two most frequent pathological entities leading to CA, asphyxia and VF, differ significantly with regard to metabolic disturbances. Although further studies are required to fully elucidate the role of these metabolic changes, our findings may be useful in the identification of novel diagnostic and prognostic biomarkers.

## Materials and Methods

The experimental protocol was approved by the Greek General Directorate of Veterinary Services (reference number 3532/04-06-2014). The study was performed according to Utstein-style guidelines on 20 healthy Landrace/Large-White pigs of female sex, aged 9 weeks and with an average weight of 20 ± 2 kg. The number of the animals was based on the guiding principles underpinning the humane use of animals in scientific research (replacement, reduction, refinement - three “Rs”). The animals were fasted overnight but had free access to water. Prior to any procedure, animals were randomized into two groups with the use of a sealed envelope indicating the animal assignment to either the ACA group (n = 10) or the VFCA group (n = 10). The investigators were blinded to the group allocation during the experiment and when assessing the outcome.

### Experimental protocol

All animals were prepared as described in the supplementary information. Baseline data were collected after allowing the animals to stabilize for 60 minutes. Asphyxia was induced by clamping the endotracheal tube at the end of exhalation while ACA was confirmed when MAP < 30 mmHg, as previously described^29^. VFCA was induced using a pacing wire, as previously described, while CA was confirmed electrocardiographically and by a drop in MAP < 30 mmHg^30^. Immediately following confirmation of CA, mechanical ventilation and drugs were ceased. Blood samples were collected from the internal jugular vein at 1 min intervals after the onset of asphyxia and VF in the ACA and VFCA group, respectively.

After 5 minutes of untreated CA, CPR was started according to the 2010 European Resuscitation Council Guidelines on Resuscitation^31^. Mechanical ventilation was resumed with 100% oxygen, while chest compressions were maintained at a rate of 100 min^−1^ with equal compression-relaxation duration (LUCAS, Jolife, Lund, Sweden). Defibrillation was attempted with 4 J/kg monophasic waveform shock (Schiller Medical, Defigard 3002).

Endpoints were ROSC or asystole. ROSC was defined as the presence of an organized cardiac rhythm with a MAP > 60 mmHg for a minimum of 10 minutes. The surviving animals were monitored for 4 hours, while anesthesia was maintained. Blood samples were taken at 1 hour intervals during the post-resuscitation period. After the 4-hour period, anesthetics were discontinued, all catheters were surgically removed and the animals were extubated as previously described^30^.

A standardized neurology score was used for assessing neurological status of the surviving animals 24 hours after ROSC^30^. Then, a final blood sample was collected, and the animals were humanely euthanatized by an intravenous dose of thiopental (2 g). Blood samples were collected in Li-heparin tubes and centrifuged at 5,250 g and 4 °C for 10 minutes. Plasma samples were divided in aliquots of 1 ml and mixed with 10 µl of a 10% w/w aqueous solution of sodium azide (NaN_3_, Sigma-Aldrich, Italy) in order to avoid bacterial growth and were immediately stored at −80 °C until ^1^H NMR and LC-MS/MS metabolomics analyses.

### Metabolomics analyses

A total of 380 plasma samples, collected during the different phases of the experiment (1 withdrawal per minute), were analysed using ^1^H NMR and LC-MS/MS. In particular, ^1^H NMR was used to obtain a global metabolomics profile of the plasma samples and LC-MS/MS was used to exactly quantify selected sets of key metabolites, such as aminoacids, acylcarnitines, Krebs cycle intermediates, involved in cellular energy metabolism and in amino acid metabolism/catabolism. The two analytical techniques were considered useful as they complement each other.

### ^1^H NMR sample preparation and analysis

All plasma samples were gently thawed in ice, centrifuged at 15,500 g and 4 °C for 10 minutes to remove any particulate. Plasma samples were deproteinized by centrifugation for 25 min at 15,500 g and 4 °C, using 10 kDa centrifugal filter units (Amicon-10kDa; Merck Millipore, Darmstadt, Germany). Prior to filtration, the filters were washed out from glycerol by adding 500 μl of distilled water and by centrifuging for 10 min at 12,750 g at room temperature for 15 times. For the NMR analysis, 300 μl of each filtered plasma sample were diluted with 400 μl of a 0.09 M phosphate buffer solution (pH = 7.4) in D_2_O (99,9%, Cambridge Isotope Laboratories Inc, Andover, USA) containing the internal standard sodium 3-(trimethylsilyl)propionate-2,2,3,3,-d_4_ (TSP, 98 atom % D, Sigma-Aldrich, Italy) at a 0.21 mM final concentration, and transferred into 5 mm NMR tubes.

All ^1^H NMR experiments were carried out on a Varian UNITY INOVA 500 spectrometer (Agilent Technologies, CA, USA) operating at 499.839 MHz. Spectra were acquired at 300 K using the standard 1D-NOESY pulse sequence for water suppression with a mixing time of 1 ms and a recycle time of 3.5 s. Spectra were recorded with a spectral width of 6,000 Hz, a 90° pulse, and 256 scans. Prior to Fourier transformation the free induction decays (FID) were multiplied by an exponential weighting function equivalent to a line broadening of 0.5 Hz and zero-filled to 64 K. All spectra were phased and baseline corrected using the MestReNova software (Version 9.0, Mestrelab Research S.L.). Chemical shifts were calibrated using the TSP single resonance at 0.00 ppm. Two-dimensional ^1^H-^1^H COSY spectra were acquired with a spectral width of 6,000 Hz in both dimensions, 4,096 data points and 512 increments with 64 transients per increment. Assignment of the NMR resonances was performed using literature data^32^, and spiking the samples with standard compounds.

### LC-MS sample preparation and analysis

Amino acids and acylcarnitines were quantified using two kits containing lyophilized deuterated standards (Cambridge Isotope Laboratories). 20 µL of each plasma sample were spotted on Guthrie cards (made of S&S 903 filter paper; Schleicher and Schϋll, Dassel, Germany) and allowed to dry at room temperature. Each card was punched with a Wallace Autopuncher (Perkin Elmer, USA) and a dry plasma spot of 3.2 mm of diameter containing 1.7 µL of plasma was obtained. Samples were prepared as previously described^33,34^.

Succinic and malic acid were quantified using a standard working solution containing 40 µmol/L of each corresponding deuterated standards (Cambridge Isotope Laboratories) in MS-grade water. 20 µL of plasma were mixed with 20 µL of the internal standard and 300 µL of CH_3_CN/H_2_0 solution (70:30, 0.1% HCOOH) were added for the protein precipitation; the mixture was then vortexed and centrifuged at 18,900 g for 5 minutes. The supernatant was transferred to a 96 microplate and evaporated under nitrogen stream at 40 °C. The residue was suspended in 250 µL of water with 0.1% of formic acid, stirred for 5 minutes and analysed by LC-MS/MS.

α-ketoglutaric acid and pyruvic acid were quantified using a standard working solution containing 40 µmol/L of ^2^H_6_-α-ketoglutaric acid and 100 µmol/L of ^2^H_4_-pyruvic acid (Sigma-Aldrich) in CH_3_CN. Hypoxanthine and choline were quantified using a CH_3_CN solution of ^2^H_9_-choline:HCl (50 µM) and ^2^H_3_-hypoxanthine (300 µM) (Cambridge Isotope Laboratories). The sample treatment for the quantitative analysis of α-ketoglutaric acid, pyruvic acid, hypoxanthine and choline was the same, even though the two organic acids were analysed separately: a total of 10 µL of both plasma and the corresponding internal standard mixture were spiked into 300 µL CH_3_CN/H_2_0 (70:30, 0.1% HCOOH). After vortexing and centrifugation at 18,900 g for 5 minutes the supernatant was ready for analysis.

Sample analysis was performed on a HPLC Agilent 1260 Infinity (Agilent Technologies, USA) instrument coupled with a triple-quadrupole mass spectrometer API 3200 Q-trap equipped with a ESI Turbo IonSpray source (AB SCIEX, Framingham, USA). The procedure for the optimization of the chromatographic conditions is described in the Supplementary Information.

Regarding the analysis of succinic acid and malic acid the best chromatographic separation was obtained by maintaining the column at room temperature; H_2_O buffered with 10 mM of ammonium formate (NH_4_COOH) at pH 2.85 and CH_3_OH (0.1% HCOOH) were used as eluents. Samples were kept at 8 °C in Autosampler before injection. A 5 µL volume was injected via HPLC autosampler in isocratic condition: 95:5 H_2_O/CH_3_OH at a flow rate of 250 µL/min with a run time of 6 min. In negative ionization mode the ESI source temperature was set at 450 °C. By this method the found Isobaric interferences succinic acid/methylmalonic acid (m/z 117/73) was chromatographically well separated (retention times of 1.18 and 1.96 minutes respectively).

For the quantification of pyruvic acid and α-ketoglutaric acid the column was kept at room temperature; CH_3_CN and H_2_O were used as eluents for the cromathographic separation. Samples were kept at 8 °C in autosampler before injection. A 5 µL volume was injected via HPLC autosampler in isocratic condition: 70:30 CH_3_CN:H_2_O with 0.1% HCOOH at a flow rate of 450 µL/min with a run time of 4.50 min. In negative ionization mode the ESI source temperature was set at 400 °C.

Column temperature, eluents, autosampler temperature and injection volume were the same also for hypoxanthine and choline. For these analytes the isocratic conditions were: 60:40 CH_3_CN:H_2_O with 0.1% HCOOH at a flow rate of 300 µL/min with a run time of 3.50 min. In positive ionization mode the ESI source temperature was set at 450 °C.

### NMR data pre-processing

All ^1^H NMR spectra (from 0.80 to 9.00 ppm) were reduced into spectral regions (bins) of 0.01 ppm width by using MestReNova. A small bin width could be used since NMR resonances were precisely aligned due to efficacious sample buffering. The region between 4.66 and 5.18 ppm was excluded from the analysis as it shows artefacts arising from water signal suppression, and bins corresponding to drug signals were also removed. A total of 384 bins were obtained. The integrated area within each bin was normalized to a constant sum of 100 for each spectrum in order to minimize the effects of variable concentration among different samples. The plasma samples of one ACA animal exhibiting several high intensity resonances likely due to an exogenous contaminant were excluded from the analysis.

### Statistical data analysis

Multivariate data analysis based on projection methods was applied to NMR data both for exploratory data analysis and for data modelling. Specifically, Principal Component Analysis (PCA) was used to discover outliers and to recognize peculiar patterns in the collected data while Projection to Latent Structures regression (PLS) was applied to model the time evolution of the metabolite content of the samples and to drive discriminant analysis (DA). To improve model interpretation, PLS models were post-transformed into the equivalent ptPLS2 model where the predictive and the orthogonal part of the latent space were identified^35^. Multivariate Batch Statistical Process Control (BSPC)^11^ after linear expansion of the timescale was applied to investigate the trajectories of the plasma samples during the experiment. According to good practice for model building, models were validated through 7-fold full cross-validation, as well as through permutation test on the response (1000 random permutations). Models were refined by Variable Influence of Projection (VIP) selection in order to maximize Q^2^ (i.e. the cross-validated R^2^). Stability selection based on Monte-Carlo sampling (50 subsets were extracted with prior probability equal to 0.70) and PLS-VIP based was applied to select the most important variables used in data modelling^36^. Data were mean centered and Pareto scaled prior to perform data analysis.

Clinical data were expressed as mean ± standard deviation (SD) for continuous variables and as percentages for categorical ones. The Shapiro-Wilk test was applied for normality analysis of the parameters. The comparison between groups was performed using the Student’s t-test. Statistical analysis on quantitative LC-MS/MS data was based on unpaired Mann-Whitney U-test for inter-groups comparison or paired Wilcoxon t-test and on mixed-effects model for within group comparison to take into account the dependence of the measurements on the same animal. A p-value equal to 0.05 was considered as the cut-off point for statistical significance. The results of the false discovery rate correction procedure were expressed in terms of q-value.

Statistical data analysis was performed by SIMCA 13 (Umetrics, Umea, Sweden), GraphPad Prism 6.0 (graphPad Software, La Jolla, CA), SPSS 17.00 (Statistical Package for the Social Sciences, SPSS Inc., Chicago, Ill., USA) and the platform R 3.0.2 (R Foundation for Statistical Computing).

### Data availability

All raw data are available upon request.

## Electronic supplementary material

Supplementary information
