# Supplementary material for: Metabolomics profiling reveals different patterns in an animal model of asphyxial and dysrhythmic cardiac arrest
Source: Sci Rep. 2017 Nov 29;7:16575. doi: 10.1038/s41598-017-16857-6 (PMC5707403; doi:10.1038/s41598-017-16857-6)
Supplement: Supplementary file 1 — Supplementary information [file 41598_2017_16857_MOESM1_ESM.doc]

**Metabolomics profiling reveals different patterns in an animal model of asphyxial and dysrhythmic cardiac arrest**

Dimitrios Varvarousis MSc^1^, Theodoros Xanthos PhD^2^, Giulio Ferino PhD^3,4^, Antonio Noto PhD^5^, Nicoletta Iacovidou PhD^6^, Massimo Mura BSC^4^, Paola Scano PhD^7,8^, Athanasios Chalkias PhD^1,9^, Apostolos Papalois PhD^10^, Fabio De-Giorgio MD^11^, Alfonso Baldi MD^12^, Paolo Mura MD^3^, Chryssoula Staikou PhD^6^, Matteo Stocchero PhD^13^, Gabriele Finco MD^3^, Ernesto d'Aloja PhD^3^, Emanuela Locci PhD^3^

^1^ MSc “Cardiopulmonary Resuscitation”, Medical School, National and Kapodistrian University of Athens, Athens, Greece

^2^ European University Cyprus, Nicosia, Cyprus

^3^ Department of Medical Sciences and Public Health, University of Cagliari, Cagliari, Italy

^4^ Metabolic diseases Laboratory, Children Hospital “A. Cao”, Cagliari, Italy

^5^ Department of Surgical Sciences, University of Cagliari, Cagliari, Italy

^6^ Aretaieio Hospital, National and Kapodistrian University of Athens, Athens, Greece

^7^ Department of Chemical and Geological Sciences, University of Cagliari, Cagliari, Italy

^8^ Institute for the Study of Macromolecules, ISMAC, National Council of Research, Lab, NMR, Milan, Italy

^9^ Hellenic Society of Cardiopulmonary Resuscitation, Athens, Greece

^10^ Experimental-Research Center ELPEN Pharmaceutical, Athens, Greece

^11^ Public Health Institute, Catholic University of Rome, Rome, Italy

^12^ Department of Environmental, Biological and Pharmaceutical Sciences and Technologies, Second University of Naples, Caserta, Italy

^13^ S-IN, Soluzioni Informatiche S.r.l., Vicenza, Italy

**Corresponding author:** Dr. Emanuela Locci, Department of Medical Sciences and Public Health, University of Cagliari, Cittadella Universitaria di Monserrato, S.S. 554 Bivio per Sestu, 09042 Monserrato (CA), Italy, Phone: +39 070 675 3918; Fax: +39 070 675 3122; e-mail: [elocci@unica.it](mailto:elocci@unica.it)

**Supplementary information**

***Animal preparation***

All animals were prepared in a standardized fashion, as previously described.^1^ Briefly, initial sedation in each animal was achieved with intramuscular ketamine (10 mg/kg), midazolam (0.5 mg/kg), and atropine (0.05 mg/kg). Propofol anesthesia (2.5 mg/kg) was also delivered as an intravenous bolus, via the lateral auricular vein.

The pigs were intubated with a 5.0 cuffed endotracheal tube. Animals were mechanically ventilated with a volume-controlled ventilator (Taema Clarys 2000, France) with end-tidal volume (15 ml/kg) and FiO2 0.21. Propofol (1 mg/kg), cis-atracurium (0.15 mg/kg), and fentanyl (4 μg/kg) were then administered intravenously to maintain adequate anesthetic depth, muscle relaxation and analgesia. Αnesthesia was maintained with Sevoflurane (Draeger Medical AG & Co, Germany), while cis-atracurium (2 μg/kg/min) was administered to ascertain synchrony with the ventilator. Additional doses of fentanyl (1 μg/kg) were administered when needed to ensure satisfactory analgesia. Respiratory frequency was adjusted to maintain normocapnoea (pCO2 35-40 mmHg) based on arterial blood gases (ABGs) analysis (Osmetech OPTI CCA-TS Blood Gas Analyzer, USA). Lactate was also measured with the same blood gas analyzer. Cardiac rhythm and heart rate were monitored by electrocardiography (ECG), using leads I, II, III, aVR, aVL and aVF (Envoy, Mennen Medical, Israel). Core temperature was maintained at 38.5 °C with a heating pad.

The left internal jugular vein and right carotid artery were surgically prepared under aseptic conditions. An arterial catheter (7 Fr, 20 cm Arrow, USA) was inserted into the aorta for measuring systolic (SAP) and diastolic (DAP) aortic pressures. Mean arterial pressure (MAP) was determined by the electronic integration of the aortic blood pressure waveform. Cardiac output (CO) was also measured and monitored (Vigileo monitor, Edward Lifesciences, Germany). A central vein catheter (7 Fr, 16 cm Arrow, USA) was inserted into the right atrium for measurement of right atrial pressures. Coronary perfusion pressure (CPP) was calculated as the difference between DAP and right atrial pressure.

^1^Varvarousi, G., *et al.* Asphyxial cardiac arrest, resuscitation and neurological outcome in a Landrace/Large-White swine model. *Lab Anim.* **45,** 184-190 (2011).

***LC-MS/MS analysis – Optimization of chromatographic conditions***

In order to find the optimal chromatographic conditions, different stationary phases were tested in preliminary studies. Best LC separation succinic acid, malic acid, hypoxanthine and choline were obtained with a Kinetex F5 (100 x 3.0 mm; 2.6 µm, 100 Å pore size) equipped with a SecurityGuard pre-column (Phenomenex, Italy), while for α-ketoglutaric acid and pyruvic acid a Poroshell 120 EC-C18 (4.6 x 50 mm; 2.7 µm, 120 Å pore size) column was used (Agilent Technologies, USA). Data were acquired and processed using Analyst 1.6 software (AB SCIEX, Framingham, USA). Chemoview 2.0.2 software (AB SCIEX, Framingham, USA) was used for the quantification of acylcarnitines and aminoacids.

Fine tuning and calibration of the instrument was made with the aim to provide the best response, focusing on resolution and mass calibration. After instrument optimization, each of the analytes and the isotopically labeled internal standards were studied by the flow injection analysis (FIA), in order to optimize the MS/MS parameters and to find the best multiple reaction monitoring (MRM) transition. The best specificity and sensitivity for their detection were thus obtained. A 10 µM solution in CH_3_CN were prepared for each compound and IS injected directly in MS without a chromatographic column, at flow rate of 10 µL/min.

***Multivariate statistical data analysis***


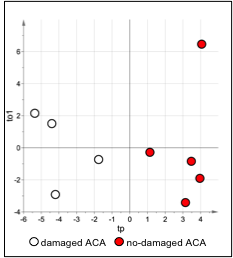


**Figure S1.** Score scatter plot of the ptPLS2-DA model of plasma samples belonging to no-damaged and damaged ACA animals at the last minute of the asphyxial period. A = 1+1 components, R^2^ = 0.90 (p-value < 0.01), Q^2^ = 0.74 (p-value < 0.01). Damaged ACA samples are represented by empty circles and no-damaged ACA samples by red circles.

**Table S1.** Metabolites accumulated at the last minute of asphyxia in damaged compared to no-damaged ACA samples, with corresponding p-values and q-values for the Mann-Whitney test, obtained merging the results of ^1^H-NMR and LC-MS/MS.

| **Metabolite** | **p-value** | **q-value** |
| --- | --- | --- |
| Lactate | <0.001 | 0.01 |
| Succinate | 0.02 | 0.03 |
| Malate | 0.03 | 0.03 |
| Hypoxanthine | 0.03 | 0.03 |
| 3-hydroxybutyrate | 0.007 | 0.02 |
| Acetone | 0.03 | 0.03 |
| Acetylcarnitine | 0.02 | 0.03 |
| Uridine | 0.01 | 0.02 |
| Tyrosine | 0.02 | 0.03 |
| Glutamate | 0.06 | 0.07 |
| Argininosuccinate | 0.06 | 0.07 |
| Phenylalanine | 0.06 | 0.07 |

**a CPR first minute b CPR last minute**

VFCA

**Figure S2.** (**a**) Score scatter plot of the ptPLS2-DA model of ACA and VFCA plasma samples at the beginning [A=1+1 components, R^2^=0.79 (p-value<0.01), Q^2^=0.66 (p-value<0.01)] and (**b**) at the end of CPR [A=1+1 components, R^2^=0.50 (p-value=0.02), Q^2^=0.32 (p-value=0.02)]. ACA samples are represented by empty circles and VFCA samples by blue triangles.

**Table S2**. Metabolites accumulated during CPR in ACA compared to VFCA samples, with corresponding p-values and q-values for the Mann-Whitney test, obtained merging the results of ^1^H‑NMR and LC-MS/MS.

| **Beginning of CPR** | | |  | **End of CPR** | | |
| --- | --- | --- | --- | --- | --- | --- |
| **Metabolite** | **p-value** | **q-value** |  | **Metabolite** | **p-value** | **q-value** |
| Lactate | 0.001 | 0.03 |  | Lactate | 0.001 | 0.05 |
| Succinate | 0.0003 | 0.03 |  | Succinate | 0.0002 | 0.02 |
| Malate | 0.006 | 0.05 |  | Inosine | 0.01 | 0.09 |
| Glutamate | 0.01 | 0.06 |  |  |  |  |
| Alanine | 0.08 | 0.15 |  |  |  |  |

Arginine is the only metabolite more represented in the VFCA group at the end of CPR (p‑value = 0.08).

**a CPR first minute b CPR last minute**

no-damaged ACA

damaged ACA

**Figure S3.** (**a**) Score scatter plot of the ptPLS2-DA model of no-damaged and damaged ACA samples at the beginning [A=1+1 components, R^2^=0.84 (p-value=0.02), Q^2^=0.73 (p-value=0.02)] and (**b**) at the end of CPR [A=1+1 components, R^2^=0.74 (p-value=0.04), Q^2^=0.62 (p-value=0.05)]. Damaged samples are represented by empty circles and no-damaged samples by red circles.

**Table S3.** Metabolites accumulated during CPR in damaged with respect to no-damaged ACA samples, with corresponding p-values and q-values for the Mann-Whitney test, obtained merging the results of ^1^H-NMR and LC-MS/MS.

| **Beginning of CPR** | |  | **End of CPR** | |  | | |  |
| --- | --- | --- | --- | --- | --- | --- | --- | --- |
| **Metabolite** | **p-value** | **q-value** | **Metabolite** | **p-value** | **q-value** |  |  | |
| Lactate | 0.01 | 0.06 | Lactate | 0.01 | 0.07 |  |  | |
| Succinate | 0.02 | 0.09 | Succinate | 0.02 | 0.09 |  |  | |
| Malate | 0.03 | 0.11 | Malate | 0.03 | 0.11 |  |  | |
| Hypoxanthine | 0.03 | 0.11 | Hypoxanthine | 0.06 | 0.15 |  |  | |
| Phenylalanine | 0.06 | 0.16 | Glutamate | 0.03 | 0.11 |  |  | |
| Fumarate | 0.01 | 0.06 | Fumarate | 0.01 | 0.07 |  |  | |
| Inosine | 0.02 | 0.09 | 3-hydroxybutyrate | 0.01 | 0.08 |  |  | |
| Uridine | 0.02 | 0.09 | Acetylcarnitine | 0.03 | 0.11 |  |  | |
| Cytidine | 0.02 | 0.09 | Tyrosine | 0.06 | 0.15 |  |  | |
| Formate | 0.02 | 0.09 |  |  |  |  |  | |

Arginine is the only metabolite more represented in no-damaged animals at the end of CPR (p-value = 0.02).

**a ACA b VFCA**

ROSC

Post ROSC 1h ACA

ROSC

Post ROSC 1h

**Figure S4.** (**a**) Score scatter plot of the ptPLS2-DA model of ACA plasma samples collected at ROSC (empty circles) and 1h post-ROSC (green circles). A=1+1 components, R^2^=0.62 (p-value=0.05), Q^2^=0.38 (p-value=0.02). (**b)** Score scatter plot of the ptPLS2-DA model of VFCA plasma samples collected at ROSC (turquoise triangles) and 1h post-ROSC (blue triangles). A=1+1 components, R^2^=0.61 (p-value=0.02), Q^2^=0.42 (p-value=0.02).

**Table S4**. Metabolites modified from ROSC to 1h post-ROSC in ACA and VFCA samples, with corresponding p-values and q-values for the Wilcoxon test, obtained merging the results of ^1^H NMR and LC-MS/MS.

| **1h after ROSC** | | | | | | | | | | |  |
| --- | --- | --- | --- | --- | --- | --- | --- | --- | --- | --- | --- |
| **ACA** | | | |  | | **VFCA** | | | | |  |
| **Metabolite** |  | **p-value** | **q-value** | |  | | **Metabolite** |  | **p-value** | **q-value** | |
| Lactate | ↓ | 0.02 | 0.04 | |  | | Lactate | ↓ | 0.02 | 0.05 | |
| Succinate | ↓ | 0.02 | 0.04 | |  | | Succinate | ↓ | 0.02 | 0.05 | |
| Malate | ↓ | 0.02 | 0.04 | |  | | Malate | ↓ | 0.04 | 0.08 | |
| Hypoxanthine | ↓ | 0.02 | 0.04 | |  | | α-ketoglutarate | ↓ | 0.02 | 0.05 | |
| Glutamate | ↓ | 0.01 | 0.03 | |  | | Hypoxanthine | ↓ | 0.02 | 0.05 | |
| Aspartate | ↓ | 0.02 | 0.04 | |  | | Glutamate | ↓ | 0.008 | 0.03 | |
| Choline | ↓ | 0.01 | 0.03 | |  | | Aspartate | ↓ | 0.008 | 0.03 | |
| Glycine | ↓ | 0.03 | 0.05 | |  | | Choline | ↓ | 0.04 | 0.08 | |
| Alanine | ↑ | 0.02 | 0.04 | |  | | Phenylalanine | ↓ | 0.03 | 0.07 | |
| Citrulline | ↑ | 0.02 | 0.04 | |  | |  |  |  |  | |
| Methionine | ↑ | 0.05 | 0.07 | |  | |  |  |  |  | |

**a Post-ROSC 1h 🡪 4h b Post-ROSC 24h**


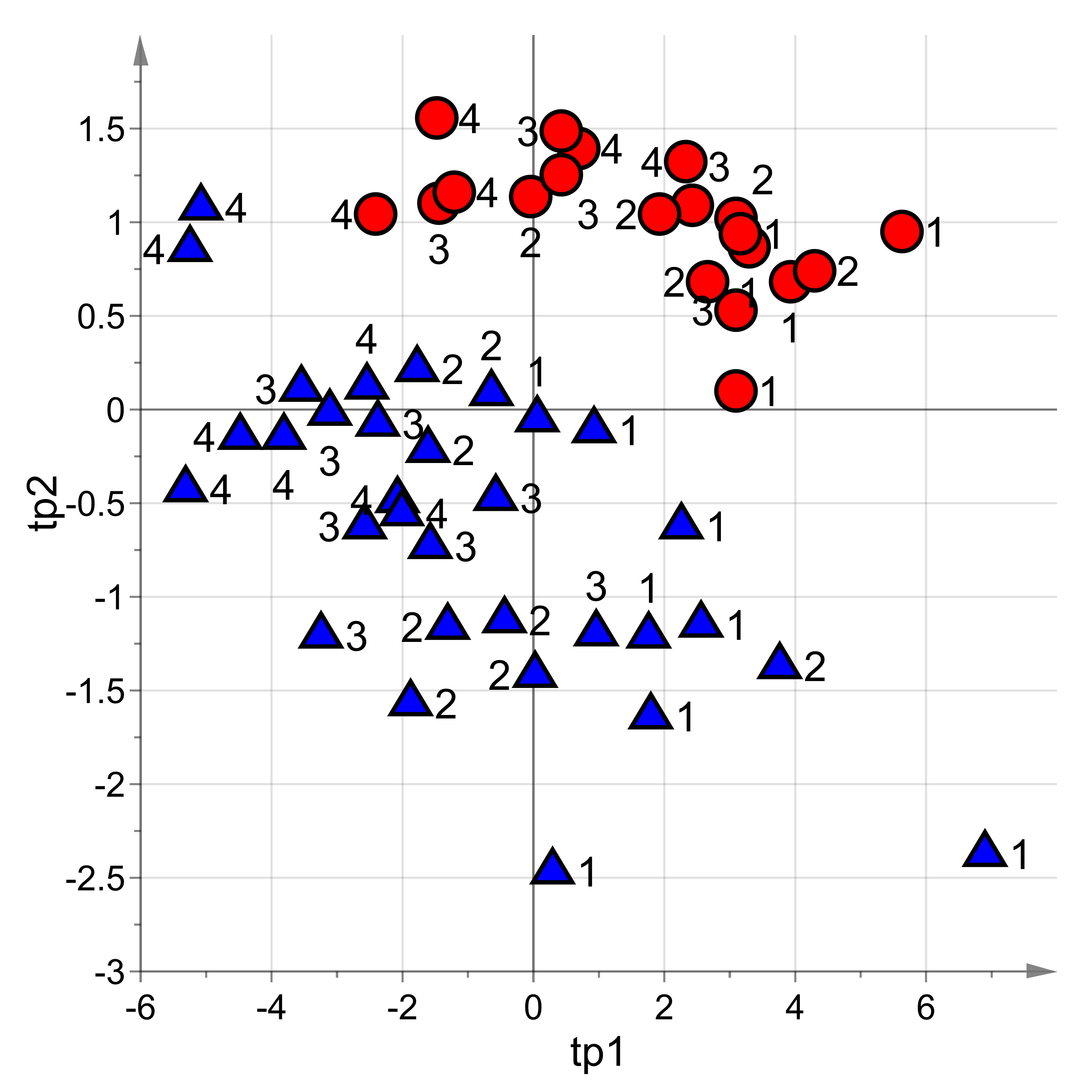

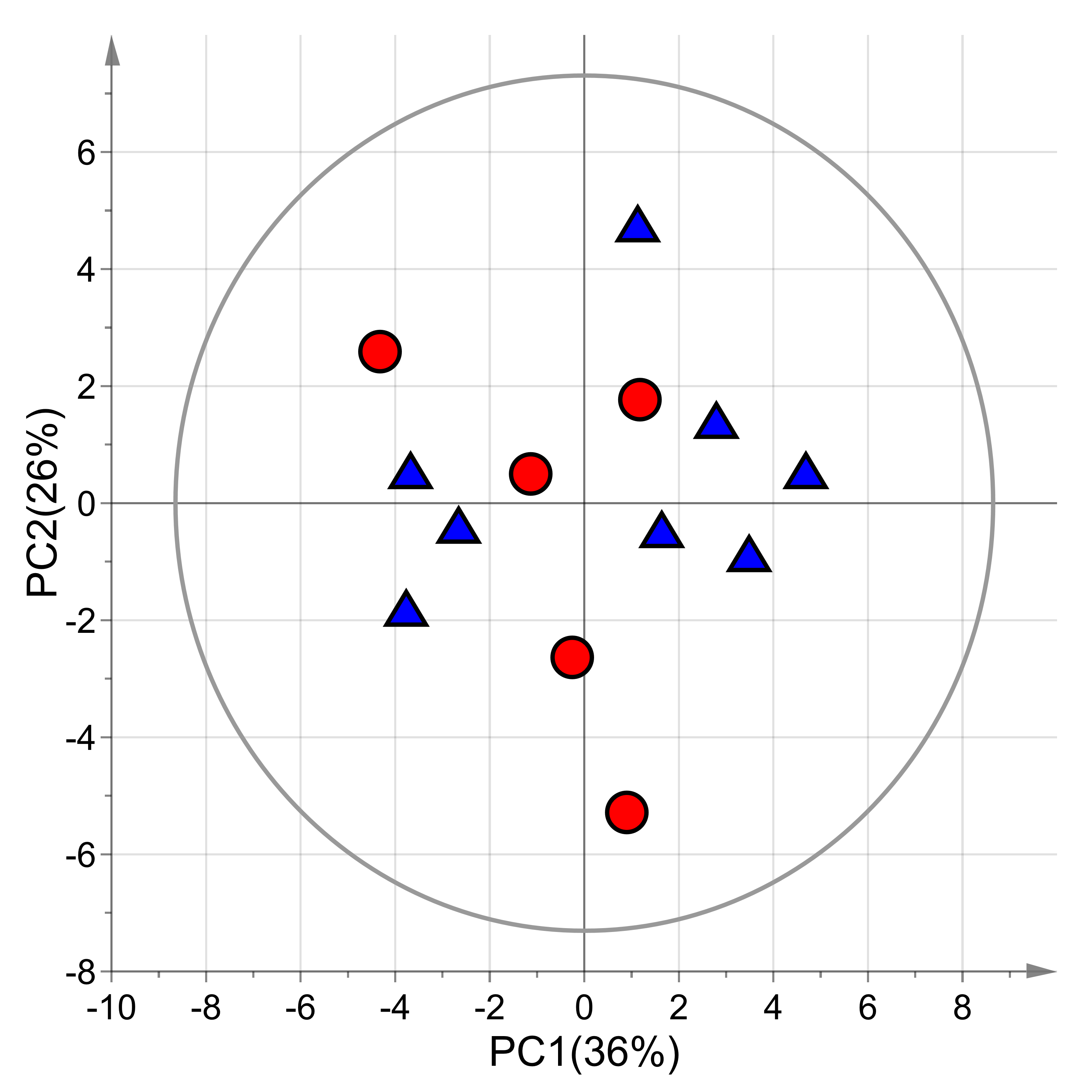


VFCA

ACA

**c** **baseline *vs*. post-ROSC 24h**


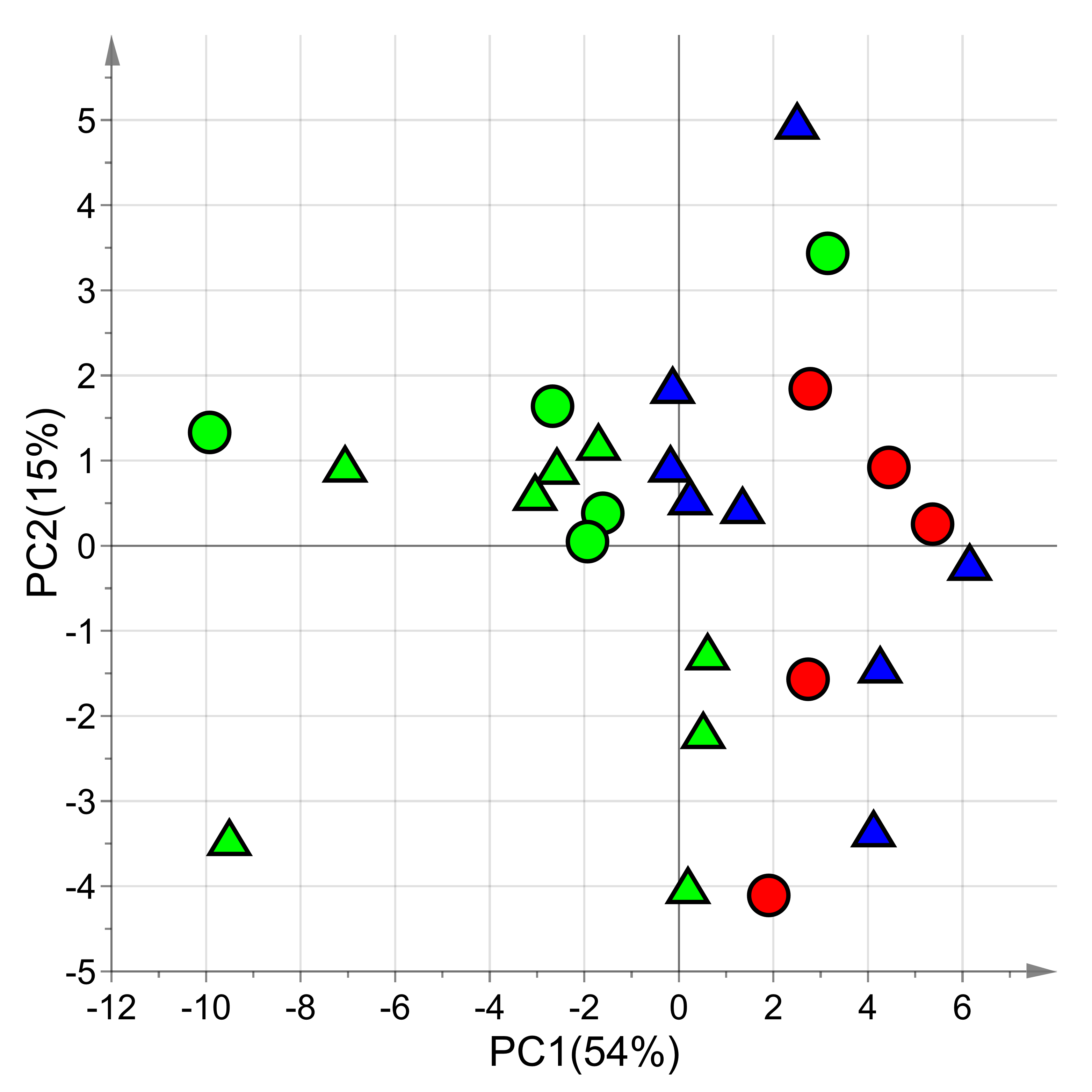


ACA and VFCA post ROSC 24h

ACA and VFCA baseline

**Figure S5**. (**a**) Score scatter plot of the ptPLS2 model of plasma samples collected during the first 4 hours post-ROSC. A = 2+3 components, for time: R^2^ = 0.58 (p-value < 0.001), Q^2^ = 0.40 (p-value < 0.001), for group: R^2^ = 0.81 (p-value < 0.001), Q^2^ = 0.64 (p‑value < 0.001). Labels are used to indicate different time points. The design matrix including time and CA cause was used as response to drive PLS regression. The predictive component tp1 mainly related to the time evolution is reported as x-axis and the predictive component tp2 explaining the differences between ACA and VFCA as y-axis. Both ACA (red circles) and VFCA samples (blue triangles) move from right to left during the first 4 hours of the ROSC phase maintaining a clear difference in their trajectories along the y-axis. (**b)** Score scatter plot of the PCA model of ACA (red circles) and VFCA (blue triangles) plasma samples collected at 24 hours post-ROSC. The explained variance of the first two components is 62%. (**c)** Score scatter plot of the PCA model of ACA (circles) and VFCA (triangles) plasma samples at baseline and 24 hours post-ROSC. The explained variance of the first two components is 69%.
